# Unilateral Oral Mucous Membrane Pemphigoid: Refractory Atypical Presentation Successfully Treated with Intravenous Immunoglobulins

**DOI:** 10.1155/2015/930859

**Published:** 2015-02-15

**Authors:** André Laureano, Jorge Cardoso

**Affiliations:** Department of Dermatology and Venereology, Hospital de Curry Cabral, Centro Hospitalar de Lisboa Central, 1069-166 Lisboa, Portugal

## Abstract

A 57-year-old male presented with a 6-month history of blisters and painful erosions on the right buccal mucosa. No skin or other mucosal involvement was seen. The findings of histopathological and direct immunofluorescence examinations were sufficient for the diagnosis of oral mucous membrane pemphigoid in the context of adequate clinical correlation. No response was seen after topical therapies and oral corticosteroids or dapsone. Intravenous immunoglobulin was started and repeated every three weeks. Complete remission was achieved after three cycles and no recurrence was seen after two years of follow-up. The authors report a rare unilateral presentation of oral mucous membrane pemphigoid on the right buccal and hard palate mucosa, without additional involvement during a period of five years. Local trauma or autoimmune factors are possible etiologic factors for this rare disorder, here with unique presentation.

## 1. Introduction

Mucous membrane pemphigoid (MMP) describes a heterogeneous group of chronic autoimmune subepithelial blistering diseases, primarily affecting mucous membranes, with or without skin involvement [1]. Although scarring is the clinical hallmark, it may not be obvious in the oral mucosa, which is the most commonly affected site. Lesions typically consist of desquamative gingivitis, erythematous patches, blisters, and erosions covered by pseudomembranes [2]. Autoantibodies binding to the epithelial basement membrane zone (BMZ) have been demonstrated in this subset, targeting bullous antigens 1 and 2, laminin 332 and laminin 311, type VII collagen, *β*4-integrin subunit, and some nonidentified basal membrane zone antigens [3, 4]. Any oral cavity location can be involved and patients usually have a good prognosis.

## 2. Case Presentation

**Figure 1:**
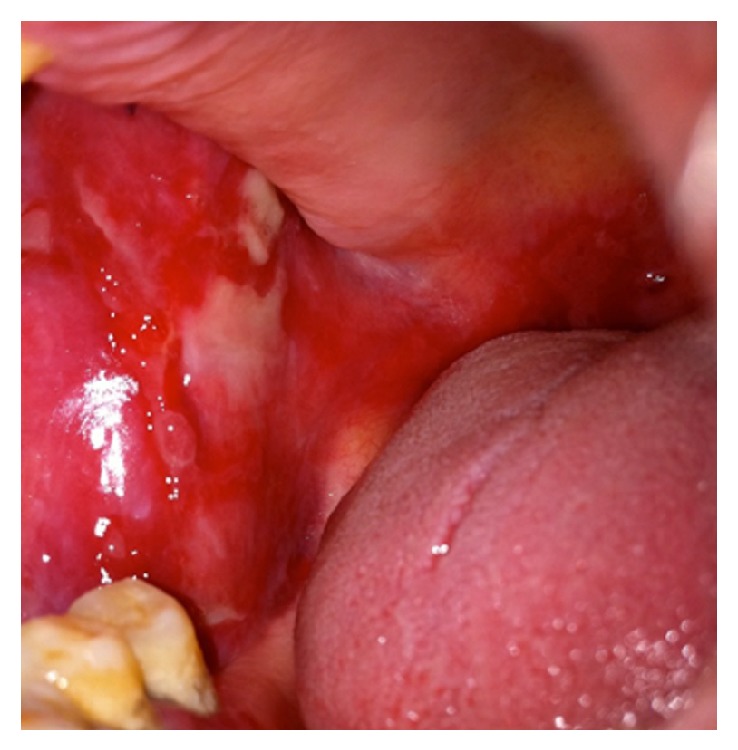
At presentation multiple painful erosions and pseudomembrane-covered erosions on the right buccal mucosa were seen.

A 57-year-old male presented with a 6-month history of blisters and painful erosions on the right buccal mucosa. His medical history was relevant for hypertension and hypothyroidism. He had been taking valsartan and levothyroxine for years and denied the use of topical drugs and previous dental procedures. On physical examination, the patient was found to have few bullae, erosions, and pseudomembrane-covered erosions on the right buccal mucosa (Figure 1).

**Figure 2:**
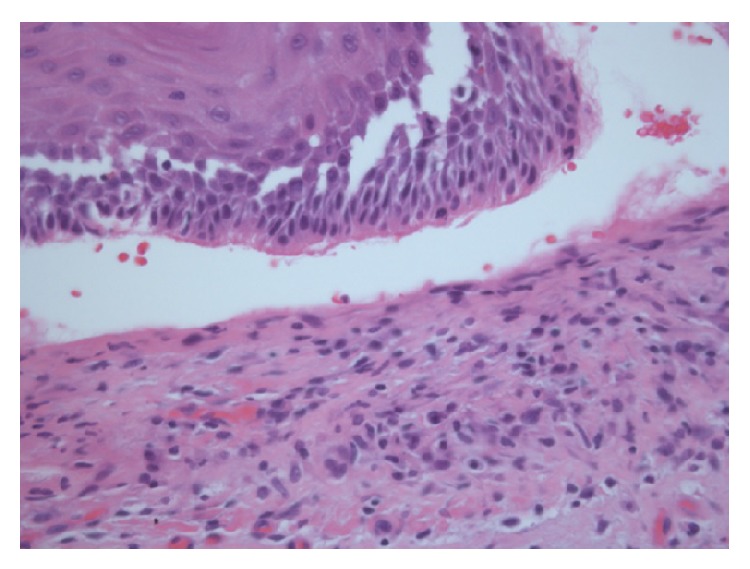
Histopathological examination of a bullous lesion revealed a subepithelial blister with a mostly lymphocytic and neutrophilic dense inflammatory infiltrate in the upper corion (hematoxylin and eosin, original magnification ×100).

No skin or other mucosal involvement was seen. He had fragmented teeth with sharp edges adjacent to the lesions. Laboratory evaluation was unremarkable. Histopathological examination of bullous lesion revealed a subepithelial blister with a mostly lymphocytic infiltrate in the upper corion (Figure 2).

**Figure 3:**
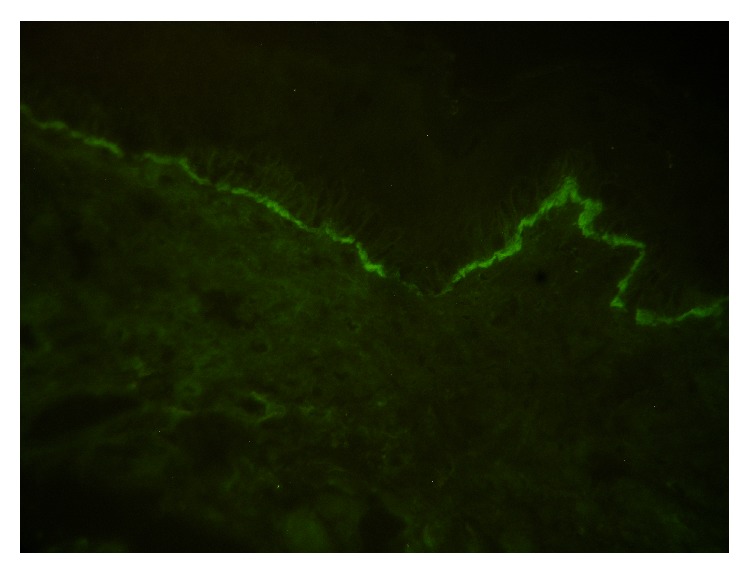
Direct immunofluorescence showed a linear band of IgG, IgA, and C3 at the epithelial BMZ (original magnification ×40).

Direct immunofluorescence of peribullous mucosa showed a linear band of IgG, IgA, and complement component 3 (C3) at the epithelial BMZ (Figure 3).

**Figure 4:**
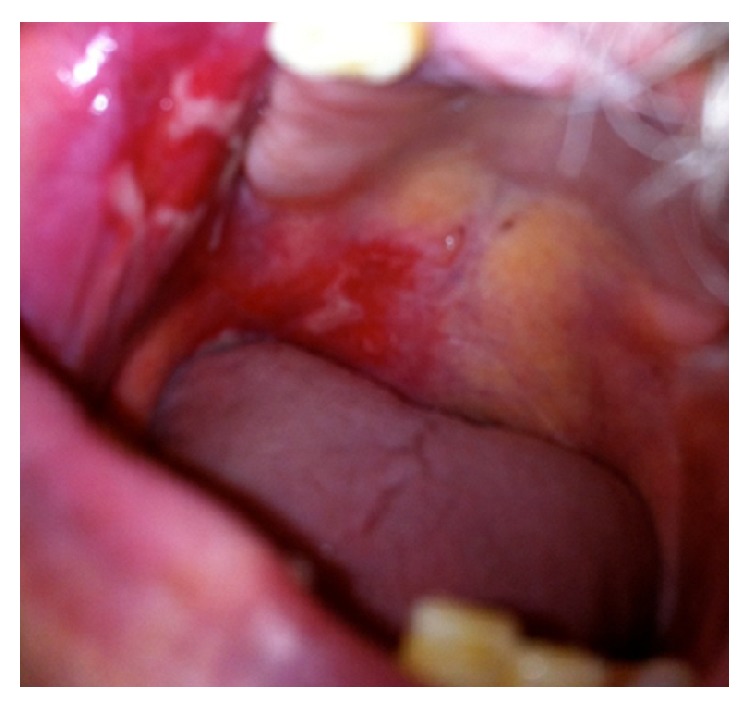
No response after topical and systemic treatment with corticosteroids and dapsone, with further involvement of the right hard palate mucosa.

ELISA was negative for antibodies against bullous pemphigoid antigens 180 and 230 and desmogleins 1 and 3. Correlation between these features allowed the diagnosis of MMP. Application of dipropionate betamethasone cream, twice daily, was started. After one year the patient had persistent bullae and erosions on the right buccal mucosa that healed without scarring. Oral prednisolone (0.5 mg/kg/d) was started for six months, and as no response was achieved, treatment with dapsone (100 mg/d) was administered during one year. Further involvement of the right hard palate mucosa occurred, erosions were extremely painful, and the patient had difficulty in eating and depression (Figure 4).

**Figure 5:**
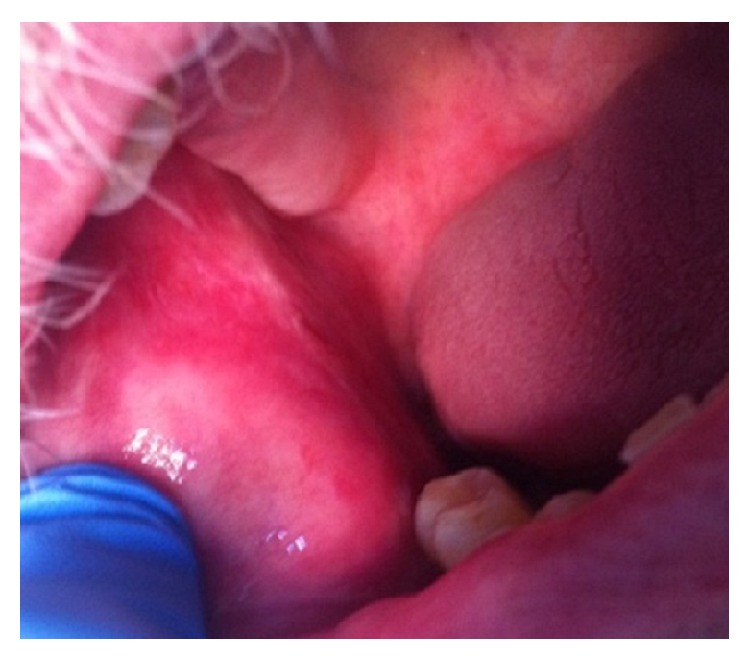
Complete response after IVIg therapy and only a delicate white pattern of reticulated scarring on the buccal mucosa had been seen after 3 years of follow-up.

Intravenous immunoglobulin (IVIg) at a dose of 2 g/kg/cycle was started and repeated every three weeks. Complete remission was achieved after three cycles. IVIg therapy was maintained for six additional months. No recurrence was seen after three years of follow-up (Figure 5).

## 3. Discussion

The findings of direct immunofluorescence were sufficient for the diagnosis of MMP in the context of adequate clinical correlation [1]. Patients with MMP with oral involvement often exhibit bilateral lesions. We reported a unilateral presentation on the right buccal and hard palate mucosa, without additional involvement during a period of five years. A possible previous chronic inflammatory process of the mucosa associated with local trauma probably exposed hidden antigens of the BMZ and evoked a secondary autoimmune response, explaining this mosaic of disease [2]. Direct immunofluorescence findings and the complete response after IVIg also suggest an autoimmune etiology, here with unique presentation [3, 5]. Since management of MMP is often difficult, our case also shows a complete response to a therapeutic option not commonly used in the limited or less severe disease.
